# Seasonal change in main alkaloids of jaborandi (*Pilocarpus microphyllus* Stapf ex Wardleworth), an economically important species from the Brazilian flora

**DOI:** 10.1371/journal.pone.0170281

**Published:** 2017-02-02

**Authors:** David Fernandes Lima, Luiza Ianny de Lima, Jefferson Almeida Rocha, Ivanilza Moreira de Andrade, Liliana Gonçalves Grazina, Caterina Villa, Liliana Meira, Leiz Maria Costa Véras, Iábita Fabiana Sousa Azevedo, Adriele Giaretta Biase, Joana Costa, Maria Beatriz P. P. Oliveira, Isabel Mafra, José Roberto de Souza de Almeida Leite

**Affiliations:** 1 Núcleo de Pesquisa em Biodiversidade e Biotecnologia, *Campus* Ministro Reis Velloso, Universidade Federal do Piauí, Parnaíba, Piauí, Brazil; 2 Faculdade de Medicina, Universidade Federal do Vale do São Francisco, Paulo Afonso, Bahia, Brazil; 3 Programa de Pós-Graduação em Biotecnologia, Rede Nordeste de Biotecnologia, Universidade Federal do Piauí, Teresina, Piauí, Brazil; 4 Grupo de Pesquisa em Ciências Naturais e Biotecnologia, Universidade Federal do Maranhão, Grajaú, Maranhão, Brazil; 5 Requimte-Laqv, Faculdade de Farmácia, Universidade do Porto, Porto, Portugal; 6 Departamento de Estatística e Experimentação Agronômica, Escola Superior de Agricultura Luiz de Queiroz – Universidade de São Paulo, São Paulo, São Paulo, Brazil; 7 Faculdade de Medicina, Universidade de Brasília, Brasília, Distrito Federal, Brazil; National Cheng Kung University, TAIWAN

## Abstract

*Pilocarpus microphyllus* Stapf ex Wardleworth (jaborandi, Rutaceae) is one of the most important Brazilian medicinal species owing to its content of pilocarpine (PIL), an alkaloid used for treating glaucoma and xerostomia. This species contains another alkaloid, epiisopiloturine (EPI), which has demonstrated effectiveness against schistosomiasis. The aim of this work was to assess seasonal changes of PIL and EPI in three populations of cultivated *P*. *microphyllus* from northeastern Brazil over one year, including the dry and rainy seasons. Alkaloid profiles were correlated to phenotypic and genetic patterns in the morphological and molecular characterizations. PIL was the primary alkaloid and its levels differed among populations in all months except September. The S01 population (green line) showed an especially high PIL content compared to populations S02 and S03 (traditional line), which had similar alkaloid contents. PIL content gradually decreased in the three populations in the rainy season.EPI content was significantly different between the green line (S01) and the traditional line (S02 and S03).S01 had a significantly lower EPI content in all months, demonstrating that it was not the best source for EPI extraction. Inter simple sequence repeat (ISSR) markers and morphological analyses clearly separated S01 from S02 and S03, in agreement with the alkaloid results. This study shows the first correlation between the chemical, morphological, and molecular markers of *P*. *microphyllus* and highlights the potential benefits of a multidisciplinary research approach aimed at supporting both industry and conservation of natural resources.

## Introduction

Jaborandi is the vernacular name of several species of medicinal plants belonging to the families Piperaceae and Rutaceae that are native to Brazil and neighboring countries [1]. In Brazil, the genus *Pilocarpus* Vahl (Rutaceae) comprises 15 species, 12 of which are endemic; most are found in the eastern part of the country at the center of the genus’ genetic diversity [2, 3, 4]. According to the literature [5, 6],*Pilocarpus* species contain many secondary metabolites, especially alkaloids. Many alkaloids have been identified, namely pilocarpine, pilosine, anhydropilosine, 3-nor-8(11) dihydropilocarpine, pilosinine, isopilocarpine, pilocarpidine, isopilocarpidine, isopilosine, epiisopilosine, epiisopiloturine, 13-nor-7(11)-dehydro-pilocarpine, *N*,*N*-dimethyl-5-methoxy-triptamine, *N*,*N*-dimethyl-triptamine, plastydesmine, (1*H)*-4-methoxy-2-quinolone, and dictamine.

*Pilocarpus microphyllus*Stapf ex Wardleworth is native to the northern and northeastern regions of Brazil and grows in eastern Pará, northwestern and northern Maranhão, and Piauí. Within the genus, leaves of this species have the highest accumulation of pilocarpine (PIL) content, which can vary from 0.5% to 1% [7]. It is one of the most important Brazilian medicinal species because PIL is used for treating glaucoma and xerostomia [8, 9]. Brazil is at present the only supplier of this ingredient for the international pharmaceutical industry,exporting tons of PIL hydrochloride and PIL nitrate every year [10].

The species is cultivated as a crop in Maranhão and Piauí, although it is still harvested from wild populations in some localities. However, since 2008, *P*. *microphyllus* has been listed as an endangered species in the Brazilian flora [11, 12].Propagation of cultivars for research and industrial applications is therefore of fundamental importance for the species’ biological conservation and for reducing pressure on wild populations. Many low-income communities depend on harvesting *P*. *microphyllus* during a particular season of the year, and companies use the species for industrial PIL extraction, so more information about the seasonality of alkaloid contents will benefit both groups.

For many years, the biological activity of most of the alkaloids found in *P*. *microphyllus*, apart from PIL, remained largely unknown. However, another alkaloid, epiisopiloturine (EPI), has been of interest in the scientific community. EPI wasfirst identified in 1978 [13] and is now considered a promising alkaloid for combatting schistosomiasis [14]. The anti-inflammatory and antinociceptive activities of EPI have been characterized [15], and chemical parameters have been improved and developed for industrial-scale isolation and spectroscopic characterization[16]. Our group is conducting other studies, such as the nanopharmaceutical application of EPI in liposome systems[17], and thermal characterization and preformulation of the prototype EPI with pharmaceutical excipients [18]. EPI is obtained from the industrial biomass waste from industrial PIL production [16], so its pharmaceutical application results both in environmental benefits and an increased economic importance of the species.

The principal aim of the present work was to identify, quantify, and evaluate seasonal changes in the two main imidazole alkaloids, PIL and EPI, in three populations (S01, S02, and S03) of cultivated *P*. *microphyllus* in the state of Piauí, Brazil, over one year, including the dry and rainy seasons. Morphological and molecular characterizations of the same populations were correlated with the alkaloid profiles to investigate the genetic diversity of cultivated collections of *P*. *microphyllus* in Piauí.

## Materials and methods

### Plant material

**Fig 1:**
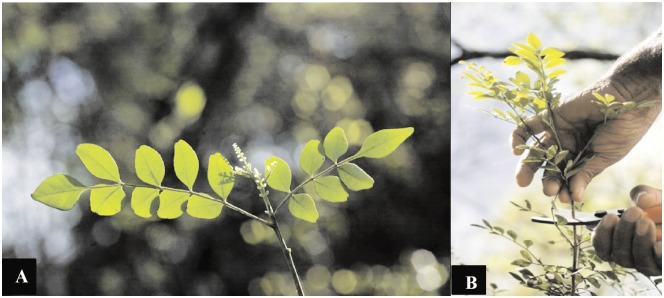
*Pilocarpus microphyllus*Stapf ex Wardleworth(jaborandi). (A) *P*. *microphyllus*, highlighting the terminal leaflets (Photo: Anidro do Brasil Extrações S.A). (B) *P*. *microphyllus* harvested with pruning shears (Photo: Anidro do Brasil Extrações S.A.).

Samples of cultivated *P*. *microphyllus*(Fig 1A) were obtained from the collection maintained at the Anidro do Brasil Extrações S.A. farm (3°6′S, 41°47′W), which is a plantation situated in the municipality of Parnaíba, Piauí state, Brazil. The company gave the permission to conduct the study on its site and it has the Certificate of Regularity—CR fromBrazilian Environment Authority IBAMA (Brazilian Institute of Environment and Renewable Natural Resources).Voucher specimens were collected and identified by Dr. Ivanilza Moreira de Andrade (Department of Biology, Federal University of Piauí) and deposited in the herbarium of the Parnaíba Delta (HDELTA) at the Federal University of Piauí (UFPI), Campus Ministro Reis Velloso, Parnaíba, Piauí, Brazil, under the numbers 2866 (S01), 2869 (S02), and 2874 (S03). The names of botanical taxa and their authors follow the*Flora do Brasil* 2020 list [4]; the description and illustrations were made from samples collected during the study.

### *Pilocarpus microphyllus* sampling and harvest

Fifteen adult plants between 0.5 and 2.0 m tall were selected and identified according to their leaf color and general morphology. The plants were categorized into three groups, each with five morphologically similar specimens: S01 (plants 1–5), S02 (6–10), and S03 (11–15). S01 represented the “jaborandi green line,” a form of *P*. *microphyllus* informally recognized as distinct within the jaborandi extractive industry. The other two groups (S02 and S03) were designated the “jaborandi traditional line.” Samples for chemical analysis were harvested between the 25^th^ and 30^th^ days of each month over one year. Young branches were harvested with pruning shears (Fig 1B). The material was dried in the sun until water content measured by an OHAUS^®^ MB45 moisture analyzer was less than 15%.

### Alkaloid extraction from *P*. *microphyllus* leaves

Dried powdered *P*. *microphyllus* leaves (5 g) were extracted with chloroform in an alkaline solution of 10% ammonia hydroxide at pH 12. This mixture was stirred for 30 minutes (Orbital Shaker, Nova ética^®^ 109 model), filtered with cotton, and partitioned with a 5% sulfuric acid solution. The acid solution was collected and the leaves were re-extracted and partitioned again. The alkaloid-rich acid solutions were homogenized and analyzed by HPLC.

### High performance liquid chromatography (HPLC) analysis

The alkaloid-rich acid solution was diluted (1:10) with the mobile phase (potassium phosphate, 5% KH_2_PO_4_, pH 2.5), filtered with a 0.45 μm pore membrane, and analyzed by HPLC (LaChron Elite^®^, L-2000 system; Merck–Hitachi, Tokyo, Japan). The column was a Merck/Lichrospher^®^ 60 RP, select B, 5 μm, 250 × 4mm, with a flow rate of 1 mL/min and an injection volume of 20 μL. The oven was set to 50°C and a UV detector was used at 216 nm. External standards were used to identify and quantify the alkaloids [16]. All solvents used in HPLC analysis were from Merck KGaA (Darmstadt, Germany).

### Alkaloid standards

PIL was isolated, purified, and provided by Anidro do Brasil Extrações S.A. Company, an international supplier of this chemical. EPI was isolated and purified at the Federal University of Piauí (UFPI), using methods reported in our previous studies [14, 16]. PIL and EPI were dissolved in acetonitrile-formic acid 1% (100 μg/ mL) and analyzed by LC-MS(AmaZon SL system, Bruker Daltonics; Bremen, Germany)to confirm their structure and purity prior to their use as standards. The conditions used for mass spectrometry detection were as follows: electrospray under positive mode and nebulizing gas flow at 2.5 L/min, interface voltage at 4.5 kV, heat block temperature at 230°C, and helium as the collision induced dissociation gas at 17 kPa.All solvents used in LC-MS analysis were HPLC-grade solvents from Merck KGaA (Darmstadt, Germany).Nuclear Magnetic Resonance spectroscopy (NMR) was done to confirm EPI alkaloid and its three stereoisomers structures. NMR experiments were carried out using a Bruker Avance III 600 HD spectrometer,operating at 150.92 MHz for carbon and 600.13 MHz for protons; it was equipped with 5 mm Prodigy CryoProbe and pulse gradient units, which are capable of producing magnetic field pulsed gradients of 50 G cm^-1^ in the z-direction. The NMR spectra were obtained at atemperature of 300 K in deuterium oxide (D2O) or methanol-d4; the chemical shifts were referenced withsodium trimethyl-silyl -[2,2,3,3-d4]-propionate (TSP) or tetra-methylsilane (TMS), respectively. Standard 1D ^1^HNMR experiments, i.e. using 30° pulses, an acquisition time of 2.7 s, a relaxation delay 1 s, and 16 transients of a spectral width of 9600 Hz, were collected into 64 K time domain points. 1H NMR experiments for the samples in water solution were performed with water suppression, using excitation sculpting with gradients, and an acquisition time of 1.7 s, a relaxation delay of 2 s, and with 16transients of a spectral width of 10000 Hz, and were collected into 32 K time domain points.

### Environmental conditions

All the sampled plants were cultivated in the same locality, under the same agronomic, irrigation, and climatic conditions (temperature, humidity, and rainfall). Rainfall was measured daily in mm with a calibrated rain gauge. The temperature (°C) and humidity (%) were recorded with a calibrated digital thermo hygrometer (Cole-Parmer^®^, EW-90080-03 model) during harvests. The harmonic means were calculated for each parameter, taking into consideration the dry and rainy seasons.

### Morphological quantitative variables

The following 11 morphological characters were measured in cm in the 15 plants: overall length and width of each parameters, imparipinnate leaf, length of the petiole, number of leaflets per leaf, length and width of the terminal leaflet blade, length and width of the terminal leaflet petiolule, length and width of one of the most distal pair of lateral leaflets, and number of secondary veins in the leaflets [19].

### Molecular markers

The DNA extraction and inter simple sequence repeats (ISSR) analysis were carried out on samples from the same 15 individual specimens measured for morphology. DNA was extracted from silica dried leaf material, following a protocol [20] modified for microtubes, using autoclaved sand and modified 2% cetyl trimethyl ammonium bromide (CTAB) buffer to disrupt the cell membranes [21]. Secondary compounds were isolated with a 24:1 mixture of chloroform to isoamyl alcohol and precipitated with isopropanol. The pellet was cleaned three times with 70% ethanol. The pellet was dissolved in 100 μL of Tris-ethylenediaminetetraacetic acid (EDTA) buffer (10 mM Tris, 1 mM EDTA). DNA quality was checked through gel electrophoresis using 1% agarose with TBE buffer (Tris-borate EDTA) and ethidium bromide at 100 V for 30 min.

Eleven polymorphic ISSR primers used were from previous studies [12] (Table 1). The samples were amplified by the polymerase chain reaction (PCR) using a modified version of the protocol [22]. The DNA extracts were diluted 10-fold to optimize amplification. The PCR conditions were carried out with a final volume of 10 μL, containing 1.0 μL of DNA extract, 7.5 pmol of each primer (Table 1), and 5 μL of TopTaq^™^ Master Mix (Qiagen Biotechnology). The Esco^®^ Swift^™^ MaxPro thermocycler program included an initial denaturing (pre-melt) at 94°C for 90 s, followed by 35 denaturing cycles at 94°C for 40 s, annealing at 47°C for 45 s, and extension to 72°C for 90 s, with an additional final cycle of 94°C for 45 s, 44°C for 45 s, and 72°C for 10 min [23].

**Table 1:** Primers used for generating polymorphic ISSR markers in *Pilocarpus microphyllus* genotypes, showing the respective levels of polymorphic loci.

Primer [repetitions]^a^	Polymorphic loci
**844 [(CT)**_**8**_ **RC]**	**14**
**CHRIS [(CA)**_**7**_ **YG]**	**13**
**DAT [(GA)**_**7**_ **RG]**	**9**
**ISSR-4 [(AC)**_**8**_ **YT]**	**9**
**ISSR-6 [(AG)**_**8**_ **YT]**	**8**
**843 [(CT)**_**8**_ **RA]**	**9**
**JOHN [(AG)**_**7**_ **YC]**	**5**
**MANNY [(CAC)**_**4**_ **RC]**	**10**
**MAO [(CTC)**_**4**_ **RC]**	**12**
**OMAR [(GAG)**_**4**_ **RC]**	**14**
**TERRY [(GTG)**_**4**_ **RC]**	**8**

^a^ Degenerate bases used: Y (C or T), R (A or G).

The amplified fragments were analyzed by electrophoresis in a 1.5% agarose gel with SB buffer (10 mM sodium hydroxide, pH 8.5 with boric acid) for 2 h at 100 V [21], stained with ethidium bromide (1.0 mg/L for 30 min), and destained in distilled water for 5 min. The agarose gel was visualized under UV light and a digital image was obtained using a photo documentation system (L-PIX, Loccus Biotecnologia). For each gel run, a negative (water) control was used to detect any contamination problems. Five to ten percent of the quantifications were randomly repeated in all cases to guarantee repeatability of the bands observed. The gels were analyzed using GelCompar II^®^ version 5.0 (Applied Maths NV, Saint-Martens-Latem, Belgium) to align the bands according to marker and to identify the fragments (200–1500 base pairs). The results were used to construct a genetic binary matrix in which each cell was assigned with presence (1) or absence (0) of a given fragment (band). This matrix was used for further analysis.

### Data analysis

#### Chemical analysis

All alkaloid assay data were expressed in percentages (%, w/w) as mean values ± standard deviation. The chemical results were checked for normality and homoscedasticity using the Shapiro-Wilk and Bartlett tests, respectively. When the assumptions were not met, the nonparametric Kruskal-Wallis test was used, followed by the Dunn test for multiple comparisons. The Tukey test was used to evaluate the statistical differences within and among samples. Differences were considered significant when *p*< 0.05. All statistical analyses were performed with the ExpDes.pt package in R software version 3.2.3, and with SAS 9.3 software [24]. Graphics were prepared using R [25] and OriginPro 8.5 [26].

#### Morphological analysis

Principal coordinate analysis (PCoA), using Euclidean distances between individuals computed from the matrix of 11 quantitative morphological variables, was used to explore the similarity relationships between individuals. A principal component analysis (PCA) was used to visualize the major trends of morphological variation in the data set. The “broken stick” test was applied to determine which principal components were significant in the analysis.

Linear discriminant analysis (LDA) was used to analyze the same quantitative morphological matrix but with the fifteen individuals classified into two groups, one consisting of the "green" population S01 and the other consisting of the two "traditional" populations S02 and S03. For this analysis the columns of the data matrix (the variables) were standardized to zero mean and unit variance. All analyses were conducted with PAST version 2.02 [27].

#### Population genetic variability and structure

The binary ISSR matrix of 15 rows and 111 columns was analyzed using GenAlEx 6.5 software [28, 29] to estimate the genetic variability within and among the three populations. Values for the following parameters were recorded: number of loci (*N*), number of exclusive loci (*NE*), proportion of polymorphic loci, mean expected heterozygosity (*He*), and Shannon Index (*I*).Analysis of molecular variance (AMOVA) [30], as implemented in GenAlEx 6.5 based on estimation of the parameter PhiPT, was used to verify the degree of genetic structure within and among the three groups; 999 permutations were used for the significance tests.

#### Morphological and molecular analyses

A matrix of pairwise Gower distances between the 15 individuals, computed from the combined matrix of 11 quantitative morphological variables and 111 molecular ISSR markers, was analyzed with PCoA to investigate the similarity relationships between the individuals. Gower’s index is a dissimilarity measure that can combine binary and quantitative variables, as implemented in PAST version 2.02 [27].

## Results

### Alkaloid standards

**Fig 2:**
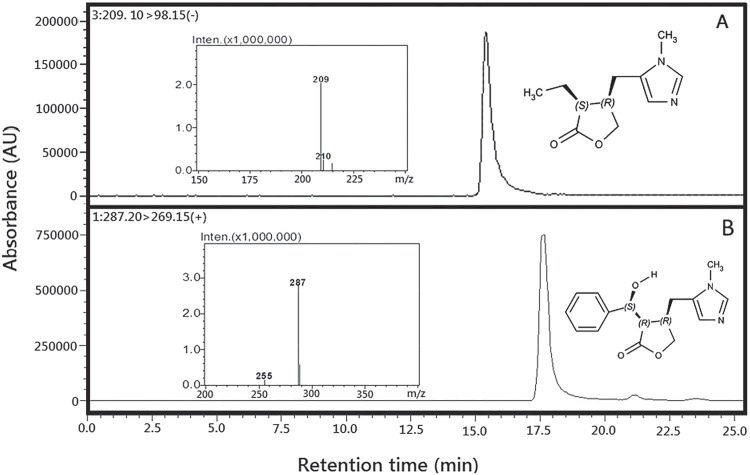
Chromatograms and mass spectra of alkaloid standards from *Pilocarpus microphyllus* analyzed by LC/MS. Pilocarpine, showing the retention time at 15.05 min and molecular weight for mass spectrometry, electron spray [M + H]^+^ at 209 Da. (B) Epiisopiloturine, showing the retention time at 17.05 min and molecular weight for mass spectrometry, electron spray [M + H] ^+^ at 287 Da.

PIL (Fig 2A) and EPI (Fig 2B) structures were confirmed by LC-MS and compared with data from the literature: PIL(3S,4R)-3-ethyl-4-[(1-methyl-1H-imidazol-5-yl)methyl]oxolan-2-one [6, 31, 32]; and EPI(3R,4R)-3-[(S)-hydroxy(phenyl)methyl]-4-[(1-methyl-1H-imidazol-5-yl)methyl]oxolan-2-one [16]. The HPLC chromatography profile of the alkaloid standards used in the seasonal study is showed in S5 Fig.The EPI alkaloid and its three stereoisomers showed in S5 Fig. had their structures confirmed by NMR. The NMR data results of these alkaloids are show in S3 Table.

### Environmental conditions

**Fig 3:**
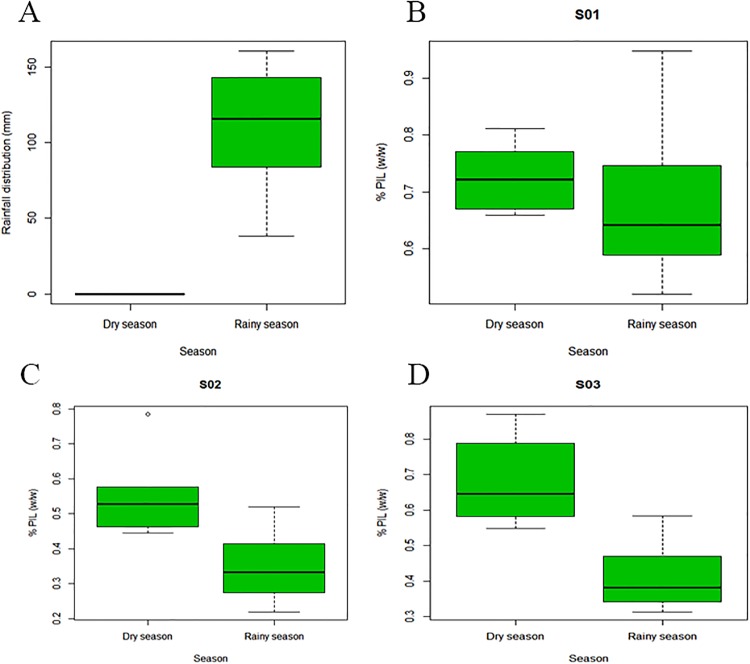
Rainfall and pilocarpine content (%) in the dry and rainy seasons. (A) Rainfall distribution (mm) during the dry and rainy seasons [0.00 mm; 109.69 mm (*p* = 0.0003), respectively] in a *P*. *microphyllus* plantation, Piauí state, northeastern Brazil. (B) Percentage of PIL (w/w) by the dry and rainy seasons [0.727; 0.683 (*p* = 0.5637), respectively] from S01. (C) Percentage of PIL (w/w) by the dry and rainy seasons [0.560; 0.350 (*p* = 0.0149), respectively] from S02. (D) Percentage of PIL (w/w) by the dry and rainy seasons [0.687; 0.415 (*p* = 0.0030), respectively] from S03.

No precipitation was recorded during the dry season from August to December (0 mm). In the rainy season (January–July), there was recorded precipitation (109.69 mm) (Fig 3A). During the harvest in the dry season, the recorded mean temperature and humidity were 32.42 ± 2.03°C and 54.08 ± 9.66%, respectively. In the rainy season, the recorded mean temperature and humidity were 33.75 ± 0.95°C and 58.97 ± 5.47%, respectively (S1 Table).

### PIL seasonality

**Fig 4:**
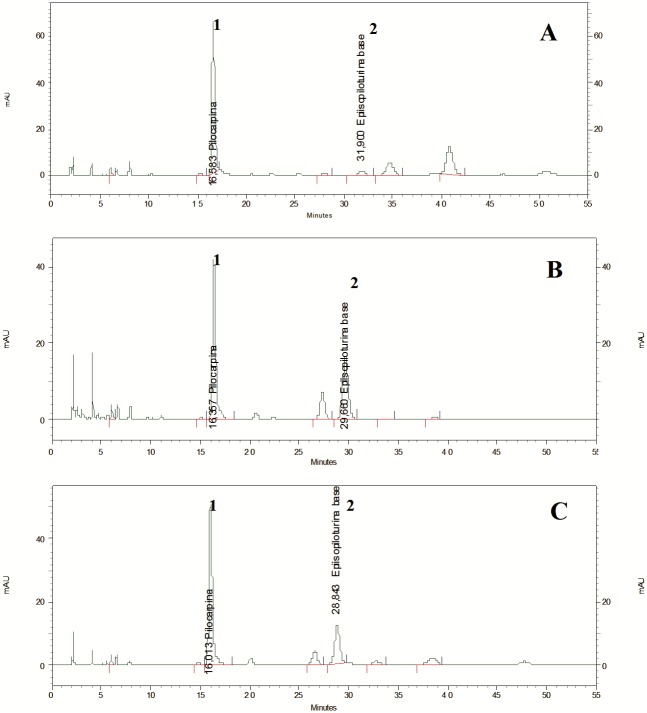
Typical chromatographic profiles obtained by HPLC analysis of the three cultivated populations of *P*. *microphyllus*. S01 group (jaborandi green line). (B) S02 group (jaborandi traditional line). (C) S03 (jaborandi traditional line). Peak no. 1: pilocarpine; peak no. 2: epiisopiloturine.

**Fig 5:**
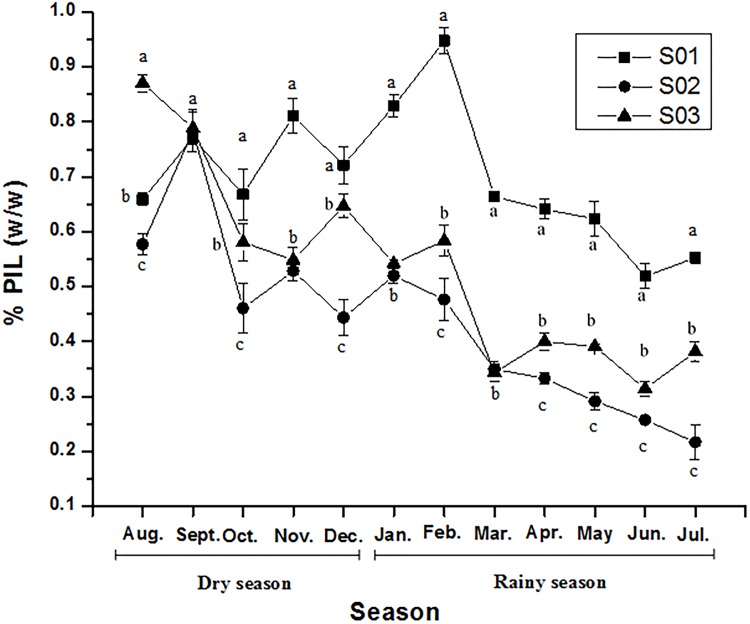
Pilocarpine (%, w/w) over one year, measured by HPLC analysis. Each point represents the mean ± standard deviation of a sample of three leaf extractions.Different letters indicate significant differences between S01, S02, and S03 with Tukey’s test at the 5% level.

The chromatogram profiles showed that PIL was the primary imidazole alkaloid in the cultivated samples (Fig 4). PIL content exhibited a significant variation between the groups (S01, S02, S03) and months according to the statistical analysis. It varied during the year and according to season and month, except in September, when the PIL contents for the three populations were the same (Fig 5).The PIL content of the three groups demonstrated normality (*p* = 0.9278) and homogeneity of variance (*p* = 0.3128) and was analyzed by time-divided installments by months. Differences among the three groups were compared using Tukey’s test, which verified that PIL content in all pairs of groups was significantly different at 5% (*p* = 0.000) (Fig 5).

The PIL content of S01 was highly significantly different from S02 and S03 using Tukey’s multiple comparisons of means (Fig 5). S02 and S03 also showed significant differences in PIL content, although they had similar chromatographic profiles (Fig 4). The S01 sample had an adequate PIL content for industrial extraction (> 0.500%) during all months, with higher contents in January, February, September, and November (Table 2). PIL levels remained constant during March, April, and May, and decreased to their lowest levels, nearly 0.500% w/w, in June and July.

**Table 2:** Pilocarpine (%, w/w) contents in S01, S02, and S03 over one year, measured with HPLC.

	S01	S02	S03
Month	PIL assay (%, w/w) ^1^		
**Aug.**	**0.659**^**d,e**^ **± 0.012**	**0.577**^**b**^ **± 0.020**	**0.870**^**a**^ **± 0.016**
**Sept.**	**0.770**^**b,c**^ **± 0.010**	**0.785**^**a**^ **± 0.038**	**0.789**^**b**^ **± 0.029**
**Oct.**	**0.670**^**d,e**^ **± 0.046**	***0*.*463***^**c,d,**^^2^ **± 0.044**	**0.582**^**c,d**^ **± 0.033**
**Nov.**	**0.811**^**b**^ **± 0.032**	**0.529**^**b,c**^ **± 0.017**	**0.548**^**d**^ **± 0.023**
**Dec.**	**0.722**^**c,d**^ **± 0.034**	***0*.*445***^**d,**^^2^ **± 0.032**	**0.647**^**c**^ **± 0.022**
**Jan.**	**0.828**^**b**^ **± 0.020**	**0.520**^**b,c**^ **± 0.013**	**0.542**^**d**^ **± 0.008**
**Feb.**	**0.947**^**a**^ **± 0.023**	***0*.*478***^**c,d,**^^2^ **± 0.038**	**0.584**^**c,d**^ **± 0.028**
**Mar.**	**0.663**^**d,e**^ **± 0.006**	***0*.*350***^**e,**^^2^ **± 0.014**	***0*.*344***^**e,f,**^^2^ **± 0.01**
**Apr.**	**0.641**^**e**^ **± 0.018**	***0*.*333***^**e,**^^2^ **± 0.010**	***0*.*400***^**e,**^^2^ **± 0.015**
**May**	**0.624**^**e**^ **± 0.032**	***0*.*291***^**e,f,**^^2^ **± 0.016**	**0.*391***^**e,**^^2^ **± 0.00**
**Jun.**	**0.519**^**f**^ **± 0.022**	***0*.*257***^**f,g,**^^2^ **± 0.007**	**0.*314***^**f,**^^2^ **± 0.014**
**Jul.**	**0.552**^**f**^ **± 0.009**	***0*.*219***^**g,**^^2^ **± 0.031**	***0*.*382***^**e,**^^2^ **± 0.018**

^1^ Data represents the mean values ± standard deviation of a sample of three leaf extractions. Different letters (a,b,c,d,e,f,g) in the same column indicate significant differences (5% probability with Tukey’s test).

^2^ The values in *italics* do not fit the criteria for industrial extraction (< 0.500% w/w).

S02 showed a significantly high PIL content only in September, and content decreased to < 0.500% in October, December, and from February to July (Table 2). S03 had a significantly high PIL content in August and September. From October to February, the PIL content was adequate for industrial extraction, while from March to July it decreased (< 0.500%), as with S02. The Tukey test did not show significant differences in the PIL content of S01 samples between the dry and rainy seasons (*p* = 0.5637) (Fig 3B). S02 and S03 showed significant differences between PIL content by season, as shown in Fig 3C and 3D (*p* = 0.0149 and *p* = 0.0030, respectively). The mean PIL values in S02 and S03 were not suitable for industrial extraction during most of the rainy season (Table 2 and Fig 3).

### EPI seasonality

The EPI content data did not meet assumptions of normality and variance homogeneity, so the nonparametric Kruskal-Wallis test was used to evaluate differences between the means, followed by Dunn’s test for multiple comparisons at the 5% significance level. EPI content differed significantly (*p* = 0.00037) among the three groups (Table 3). Dunn’s multiple comparison test showed that S01 was different from S02 (*p* = 0.0000) and S03 (*p* = 0.0017). However, no significant difference in EPI content was found between S02 and S03 (*p* = 0. 0943).

**Table 3:** Epiisopiloturine content (%, w/w) in S01, S02, and S03 over one year, measured with HPLC.

	S01	S02	S03
Month	EPI assay (%, w/w) ^1^		
**Aug.**	**0.056 ± 0.002**	**0.532 ± 0.011**	**0.216 ± 0.003**
**Sept.**	**0.056 ± 0.008**	**0.244 ± 0.017**	**0.595 ± 0.027**
**Oct.**	**0.019 ± 0.003**	**0.293 ± 0.021**	**0.155 ± 0.015**
**Nov.**	**0.040 ± 0.005**	**0.345 ±0.009**	**0.249 ± 0.018**
**Dec.**	**0.038 ± 0.004**	**0.278 ± 0.008**	**0.140 ± 0.007**
**Jan.**	**0.031 ± 0.004**	**0.353 ± 0.008**	**0.230 ± 0.005**
**Feb.**	**0.037 ± 0.003**	**0.383 ± 0.028**	**0.228 ± 0.015**
**Mar.**	**0.028 ± 0.000**	**0.286 ± 0.060**	**0.229 ± 0.061**
**Apr.**	**0.045 ± 0.002**	**0.288 ± 0.007**	**0.156 ± 0.014**
**May**	**0.023 ± 0.002**	**0.331 ± 0.025**	**0.273 ± 0.001**
**Jun.**	**0.037 ± 0.003**	**0.316 ± 0.007**	**0.284 ± 0.016**
**Jul.**	**0.044 ± 0.007**	**0.302± 0.058**	**0.333 ± 0.045**
**TMC** ^2^	**0.038** ^**c,α**^**± 0.011**	**0.330** ^**a,β**^**± 0.072**	**0.258** ^**b,β**^**± 0.116**

^1^ Data represent the mean values ± standard deviation of three leaf extractions. Mean values followed by the same letter (a,b,c) are not different at 5% significance level with the Kruskal-Wallis test (*p* = 0.00037). For Dunn’s multiple comparison test, means followed by the same Greek letter (α, β) are statistically equal at 5% significance level. S01 differed significantly from S02 (*p* = 0.0000) and S03 (*p* = 0.0017). No significant difference was found between S02 and S03 (*p* = 0. 0943) with Dunn’s multiple comparison test.

^2^ TMC: total mean contents.

### Molecular analysis

#### Intrapopulation diversity and genetic relationships

The 11 ISSR markers (Table 1) generated 111 loci (*N*) from S01, S02, and S03, with 8–12 obtained per primer pair (average of 10.9 loci/primer pair). The mean percentage of polymorphic loci (*P*) was 57.06% (Table 4). The population with the greatest genetic variability was S03 (*P* = 67.56%, *He* = 0.274, *I* = 0.400), followed by S01 (*P* = 53.15%, *He* = 0.203, *I* = 0.302). All populations showed at least one exclusive (private) band (S2 Table; S1 Fig).

**Table 4:** Genetic variation of the 15 individuals from the three groups of *Pilocarpus microphyllus* (S01, S02, S03) analyzed with GenAlEx 6.5 software.

Population	*%P*^a^	*N*^b^	*Na*^c^	*Ne*^d^	*I*^e^	*He*^f^	*uHe*^g^
**S01**	**53.15**	**5**	**1.333 ± 0.075**	**1.344 ± 0.034**	**0.302 ± 0.028**	**0.203 ± 0.019**	**0.254 ± 0.024**
**S02**	**50.45**	**5**	**1.279 ± 0.077**	**1.343 ± 0.036**	**0.293 ± 0.028**	**0.199 ± 0.020**	**0.249 ± 0.024**
**S03**	**67.56**	**5**	**1.568 ± 0.065**	**1.481 ± 0.036**	**0.400 ± 0.027**	**0.274 ± 0.019**	**0.342 ± 0.024**

^a^*%P*: percentage of polymorphic loci.

^b^*N*: sample size.

^c^*Na*: number of alleles.

^d^*Ne*: number of effective alleles.

^e^*I*: information index.

^f^*He*: expected heterozygosity.

^g^*uHe*:unbiased expected heterozygosity.

Data represent the mean values ± standard deviation.

#### Genetic differentiation between populations

The AMOVA showed that most of the genetic variation (91%) was expressed within the populations, with only 9% between them. Nevertheless, the permutation test showed a significant difference in among-population variance (Table 5) The pairwise population PhiPT values (lower hemimatrix) showed significant differences between S01 and S02 plus S03 (upper hemimatrix shows *p*-values) (Table 6).

**Table 5:** Analysis of molecular variance (AMOVA) of the three groups of cultivated *Pilocarpus microphyllus*, analyzed by GenAlEx 6.5 software.

Source	Df^a^	SS^b^	MS^c^	EV^d^	PV^e^ (%)
**Among Pops**^f^	**2**	**47.467**	**23.733**	**1.620**	**9**
**Within Pops**^f^	**12**	**187.600**	**15.633**	**15.633**	**91**
**Total**	**14**	**235.067**		**17.253**	**100**
**Stat**^g^	**Value**^i^	**P**^j^**(rand ≥ data)**			
**PhiPT**^h^	**0.094**	**0.002**			

^a^Df: degrees of freedom;

^b^SS: sums of squares;

^c^MS: mean square;

^d^EV: estimated variation;

^e^PV (%): percentage of variation among populations and within populations;

^f^Pops: population;

^g^Stat: statistic used to estimate structure;

^h^PhiPT: analogue of F statistic, used for dominant genetic data to estimate the between-group/within group structure;

^i^Value: value of PhiPT;

^j^P(rand ≥ data): probability of obtaining a PhiPT value of 0.094 or greater if there were no differences between the three groups.

**Table 6:** Pairwise genetic distances (as PhiPT values) between the three groups (S01, S02, S03) of cultivated *Pilocarpus microphyllus*, based on ISSR marker data analyzed with GenAlEx 6.5 software.

Pairwise Population PhiPT Values			
	S01	S02	S03
**S01**	0.000	0.008^a^	0.007^a^
**S02**	0.126^b^	0.000	0.093^a^
**S03**	0.111^b^	0.045^b^	0.000

^a^Lower hemimatrix: PhiPT values.

^b^Upper hemimatrix: p-values from the permutation test (9,999 permutations).

#### Morphological characterization of jaborandi (*P*. *microphyllus*)

The following description of *P*. *microphyllus* combines the characteristics observed in the plants used in this study: shrub about 40 cm tall; stem winged, ridged and pubescent; stipule pubescent, 1–2 mm long; leaves alternate, 2.0–12.7 × 2.1–7.5 cm (S2 Fig), imparipinnate, rarely paripinnate, chartaceous, petiole 0.3–3.7 cm, canaliculate, pubescent; winged, canaliculate, olive green, pubescent,1.3–11.9 cm; rachis1.0–15.5 cm, pubescent; leaflets 1–11, 1.1–8.0 × 0.9–8.0 cm, opposite, sessile except for the terminal leaflet, dark green adaxially and paler green abaxially, leaflet blade chartaceous, glabrous, elliptic to narrowly ovate, apex rounded to emarginated or retuse, base asymmetric to attenuate, rachis margin entire; leaflet venation brochidodromous, midrib prominent adaxially, planar or slightly prominent abaxially, secondary veins 6–13; terminal leaflet blade ovate, elliptic, apex rounded to emarginated or retuse, base attenuate; pellucid punctate glands present on the leaflets. Inflorescence a raceme, 13.5–39.2 cm long with small greenish-yellow flowers from March to July; fruit a dehiscent white capsule.

The individuals of the three groups overlapped substantially when the first two principal components (54.8% of variance) derived from the 11 morphological variables were plotted, although principal components two and three (35.5% of variance) partially separated S01 from the other two groups. The scree plot and broken stick test (in PAST) showed that the first four components (82.7% of variance) were significant in the PCA (S3 Fig). The LDA showed a clear separation between the green (S01) and traditional (S02 and S03) lines along the discriminant axis, but the Hotelling *t*^*2*^ test was not significant (*t*^*2*^ = 74.1, *F* = 1.6, *P* = 0.4) because of the small sample size (S4 Fig). The variables contributing most to the separation were as follows: the green line had more leaflets and the terminal leaflet blade was wider with a longer and narrower petiolule, while the traditional line had a longer petiole and the petiolule of the terminal leaflet was shorter and broader.

### Correlation between morphological and molecular data

**Fig 6:**
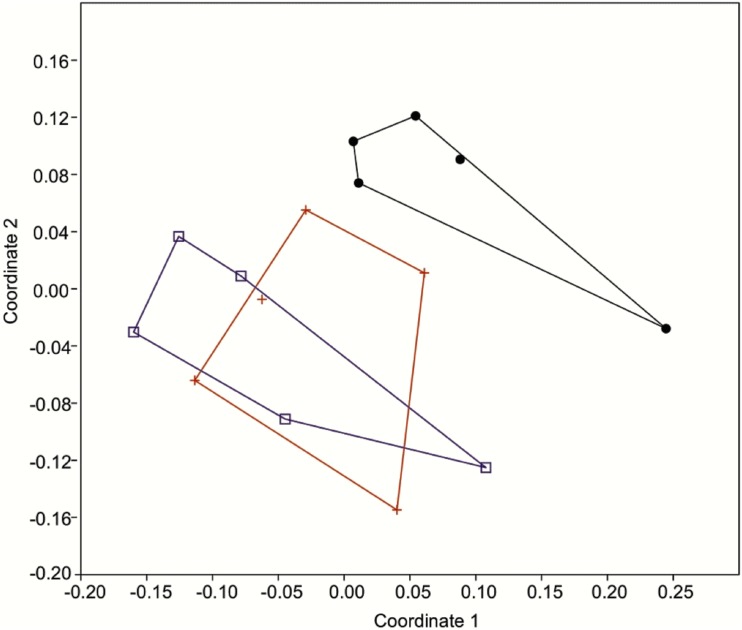
Principal coordinate analysis of 11 morphological quantitative variables and 111 ISSR binary molecular markers from three groups of *Pilocarpus microphyllus* (S01, S02, S03), representing two cultivated lines. Green line (black: S01). Traditional line (red: S02; blue: S03)

The PCoA of ISSR markers and quantitative morphological variables clearly separated S01 (green line) from S02 and S03 (both traditional line) along the first two principal coordinate axes, as shown in Fig 6. These axes represent 36.8% of the total variance.

## Discussion

This study combined several different methods to assess morphological, genetic, and chemical variation in *P*. *microphyllus*. Multivariate morphometric studies, which use statistical methods to explore the morphological variability among specimens, are frequently used to identify phenotypic differences in within species [33, 34, 35, 36, 37]. ISSR markers can reflect variation among and within small populations at relatively low costs because they are characterized by a high degree of polymorphism [38, 39]. *Pilocarpus microphyllus* has been previously studied using random amplification of polymorphic DNA (RAPD) [40] to assess germplasm genetic variability. The authors found no correlation between RAPD results for samples from wild populations and an improved genotype from Merck, which had higher dry matter and leaf area. Sandhu et al. [41] found no correlation between RAPD markers and PIL content in 20 genotypes of *P*. *microphyllus* from the state of Maranhão (Brazil). In contrast, our results showed clear genetic and phenotypic differences between the two cultivated lines, including alkaloid content. ISSR markers are known to be highly polymorphic in *P*. *microphyllus* [12] and will be informative for future studies on the genetic diversity of wild populations.

The morphological and molecular data indicated significant diversity in the *P*. *microphyllus* lines, despite the small sample sizes used in this pilot study. These findings indicate that further studies of wild populations, as well as more detailed data from currently cultivated lines, will have important applications for industrial extraction of the alkaloids and conservation of natural populations. Further investigation of genetic and phenotypic variability will be important for crossing genetically divergent parents to produce hybrids with a higher heterozygosity [33].

The major findings of this study are that PIL and EPI alkaloid contents vary by month in the dry and rainy seasons, and that in the three groups of *P*. *microphyllus* plants, alkaloid content is correlated with genetic and morphological diversity.The abundance profiles of PIL and EPI were quantified and evaluated throughout one year to assess the effects of the dry and rainy seasons. Molecular and morphometric analyses identified the genetic and morphological differences among the cultivated plants comprising green and traditional lines. Previous studies have shown that agronomic and environmental conditions can affect alkaloid contents [32], and in *P*. *microphyllus*, mineral, salt, and oxygen stresses affected the PIL content [42]. In the present study, plants were cultivated under the same environmental and agronomic conditions, and the humidity, temperature, and rainfall were monitored. All samples were harvested in the same way, using juvenile material that has been shown to yield the highest PIL levels [32].Northeastern Brazil has only two well-defined seasons: the dry season between August and December when rainfall is rarely recorded, and the rainy season between January to July when high precipitation is usually recorded [43]. For industrial use of *P*. *microphyllus*, only plants with a PIL content greater than or equal to 0.500% are considered suitable for extraction, and most wild plants have this level [7].

PIL was the primary alkaloid found in *P*. *microphyllus*, with the highest accumulation in the leaves, corroborating a previous report [41]. In this study, PIL content varied throughout the year in all samples. The lowest levels were recorded in the rainy season (Fig 3) and the highest levels in the dry season (Fig 3), suggesting that rainfall has a negative influence on PIL contenton S01 and S02 populations. An exception was found in jaborandi green line population, when rainy season just arrived there was an increasing of PIL content in January and February months (Fig 5) but from March to July it has a tendency to declineeven if this evidence was not statistically significant (Fig 3).A previous study reported that random samples of *P*. *microphyllus* harvested in the dry season showed greater PIL production [6]. These results confirm that PIL content responds to environmental conditions, especially rainfall. Local suppliers do not collect this species in the rainy season, presumably owing to the difficulty of sun drying the leaves and the rejection of raw materials with low levels of PIL below the specification for extraction. This reduction in PIL content had been previously observed in natural populations of *P*. *microphyllus* and was recorded in the cultivated lines used in this study.

Another important finding of this study is the high PIL content of the jaborandi green line (S01). This form is highly suitable for industrial PIL extraction because the PIL content is close or above the industrial specification limit of ≥ 0.500% in all months (Table 2 and Fig 5). The species is officially listed as endangered by IBAMA, the Brazilian environmental regulatory body [11], but the present study indicates that the remaining natural populations of *P*. *microphyllus* could be protected by cultivating specific lines for extractive purposes, lessening pressure on wild plants.

EPI is another alkaloid from *P*. *microphyllus* and has recently attracted attention within the scientific community because it has *in vitro* [14] and *in vivo*[44] schistosomicidal activities. It is a promising molecule for combatting a neglected disease that affects millions of people around the world, especially in underdeveloped countries [45]. EPI has an important effect against young adult *Schistosoma mansoni* parasites and inhibits egg laying. The extraction, purification, and isolation of EPI on an industrial scale, and the spectroscopic structural characterization, have been studied [16]. Other *in vivo* studies have investigated EPI’s anti-inflammatory and antinociceptive [15]activities, which might aid treatment of liver granulomas formed by *S*. *mansoni* eggs [45]. However, the seasonal content profile of EPI has not been studied previously. This alkaloid was found in significantly lower amounts in S01 in all months (Table 3), indicating that the green line is not the best source for EPI extraction. In contrast, the traditional line represented by S02 and S03 had considerably higher EPI content and would be suitable for extraction (Table 3). The jaborandi traditional line should be studied further for the industrial extraction, purification, and isolation of EPI. Despite the high EPI contents recorded from S02 and S03, only in August (S02) or September (S03) did values reach the threshold required for industrial extraction (> 0.500%), suggesting further genetic improvements or selection studies are required to enhance EPI yield.

The molecular and morphological analyses differentiated the green line individuals (S01) from those of the traditional line (S02, S03) (Fig 6, S4 Fig). In addition, the jaborandi green line has bright green leaves and a greater degree of branching, whereas the jaborandi traditional line has darker green leaves and sparser branching. The chemical data also indicated differences between the lines, with alkaloid profiles that were similar in S02 and S03 but rather different in S01 (Figs 4 and 5; Tables 2 and 3). These results indicate that two distinct forms of *P*. *microphyllus* are present in the studied plantation, perhaps deliberately selected from natural populations by early collectors. More studies are needed to fully characterize these two distinct forms of *P*. *microphyllus*.

This study shows the first correlation among the chemical, morphological, and molecular profiles of *P*. *microphyllus* and highlights the potential benefits of a multidisciplinary approach, in which the seasonal content of industrially important alkaloids can be linked to population-level genetic diversity and morphological variation. Furthermore, this study will allow better selection and development of jaborandi cultivars, with a focus on maximizing the alkaloid content. Better cultivars will reduce collecting pressure on wild populations and support year-round production of two important alkaloids.

## Supporting information

S1 Fig ISSR patterns in groups S01, S02, and S03 of cultivated *Pilocarpus microphyllus* (analyzed with GenAlEx 6.5 software). (TIF)

S2 Fig *Pilocarpus microphyllus* terminal leaflets showing petiolule. Top row: group S01 (green line) specimens 1 to 5. Middle row: group S02 (traditional line) specimens 6 to 10. Bottom row: group S03 (traditional line) specimens 11 to 15.
(TIF)

S3 Fig Principal component analysis of 11 quantitative morphological variables, showing the projection of the first two principal components. Black: group S01 (green line). Red and Blue: groups S02 and S03 (traditional line).
(TIF)

S4 Fig Linear discriminant analysis of two lines (green, traditional) of cultivated *Pilocarpus microphyllus* using eleven quantitative morphological variables. Black: group S01 (green line). Red: groups S02 and S03 (traditional line).
(TIF)

S5 Fig High Performance Liquid Chromatography profile of the alkaloid standards used in the seasonal study of *Pilocarpus microphyllus*. Peak number 1 (macaubine nitrate—0,02 mg/mL); peak number 2 (pilocarpine—0,05 mg/mL); peak number 3 (epiisopilosine—0,02 mg/mL); peak number 4 (epiisopiloturine—0,02 mg/mL); peak number 5 (isopilosine—0,02 mg/mL); peak number 6 (pilosine—0,02 mg/mL).
(TIF)

S1 Table Environmental conditions recorded during harvest of *Pilocarpus microphyllus* during rainy and dry seasons. (PI) Precipitation; (T) Temperature; (H) Humidity.
(DOCX)

S2 Table Total band patterns for binary (haploid) data by population. Data represents the mean values ± standard deviation. ^a^ No. LComm Bands: no. of Locally Common Bands; ^b^ h: diversity;^c^ uh: unbiased diversity
(DOCX)

S3 Table The NMR data results of epiisopiloturine and its three stereoisomers alkaloids epiisopilosine, isopilosine and pilosine. (DOCX)
