# Supplementary figures and images for: Seasonal change in main alkaloids of jaborandi (Pilocarpus microphyllus Stapf ex Wardleworth), an economically important species from the Brazilian flora
Source: PLoS One. 2017 Feb 2;12(2):e0170281. doi: 10.1371/journal.pone.0170281 (PMC5289444; doi:10.1371/journal.pone.0170281)

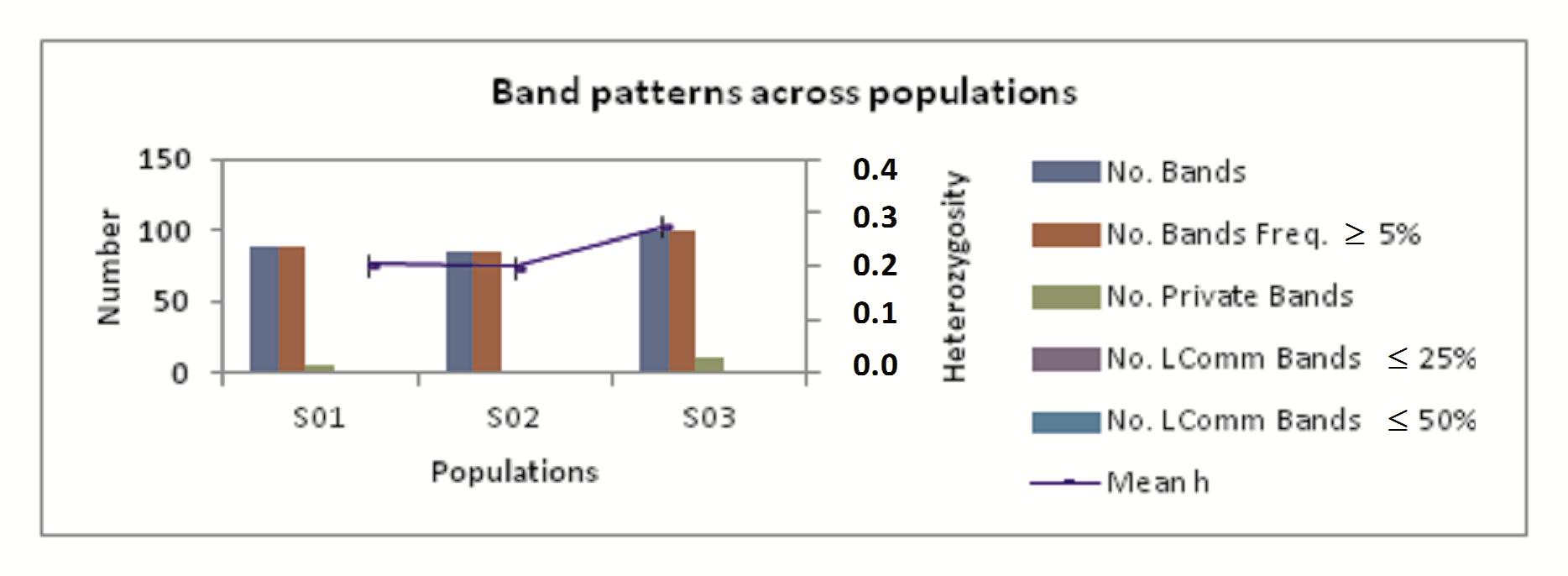

Supplement: S1 Fig — (TIF) [file pone.0170281.s001.tif]

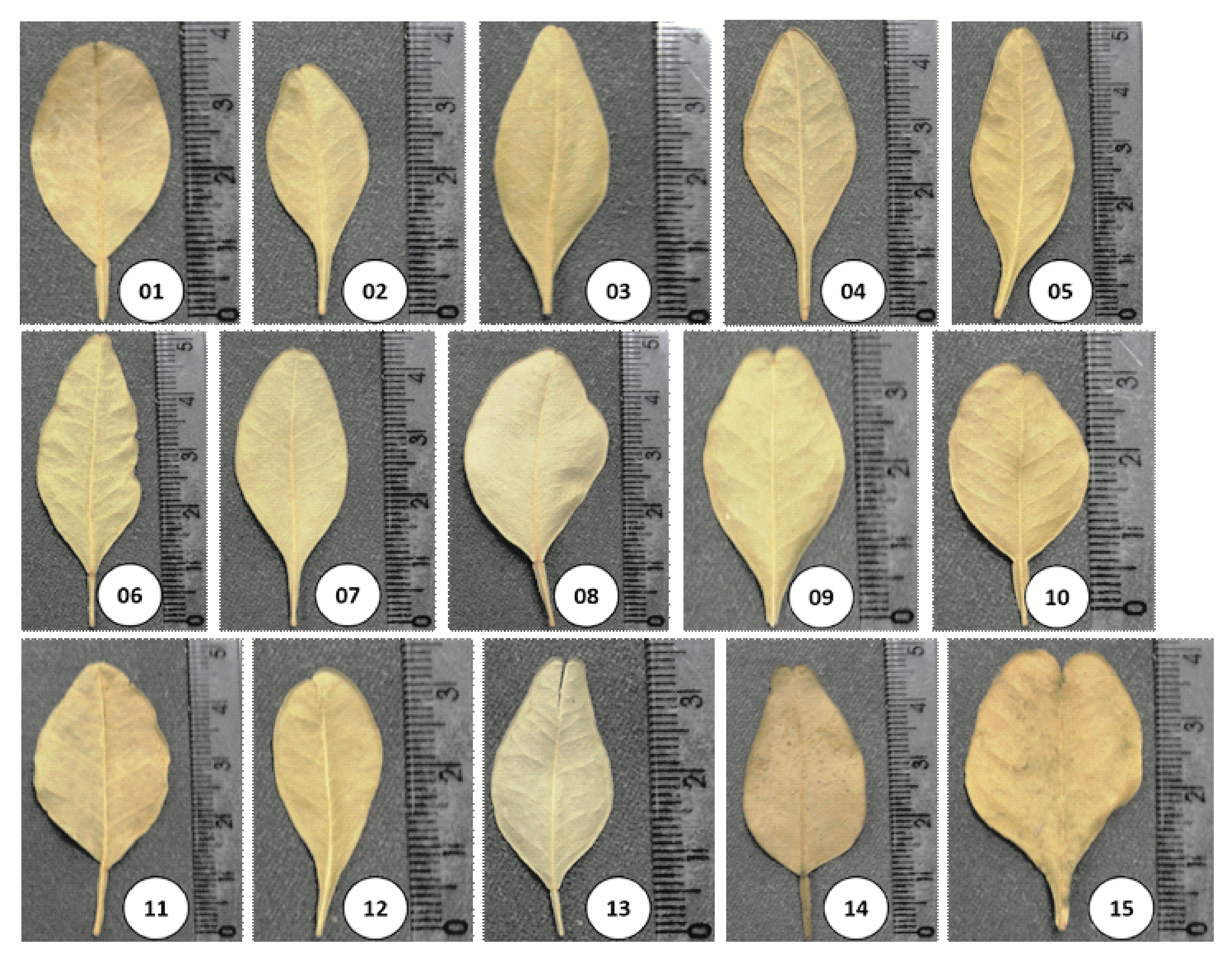

Supplement: S2 Fig — Top row: group S01 (green line) specimens 1 to 5. Middle row: group S02 (traditional line) specimens 6 to 10. Bottom row: group S03 (traditional line) specimens 11 to 15. (TIF) [file pone.0170281.s002.tif]

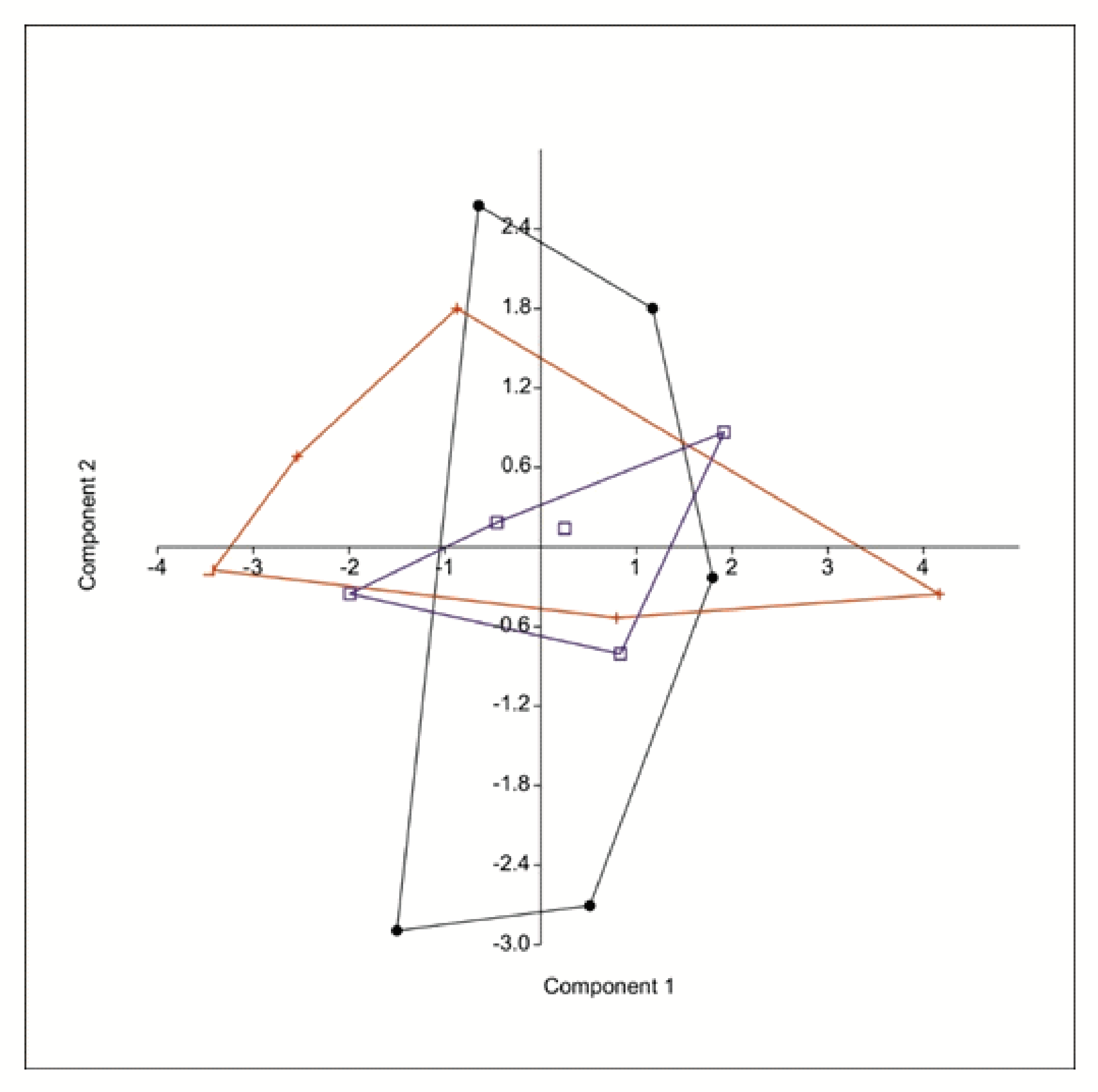

Supplement: S3 Fig — Black: group S01 (green line). Red and Blue: groups S02 and S03 (traditional line). (TIF) [file pone.0170281.s003.tif]

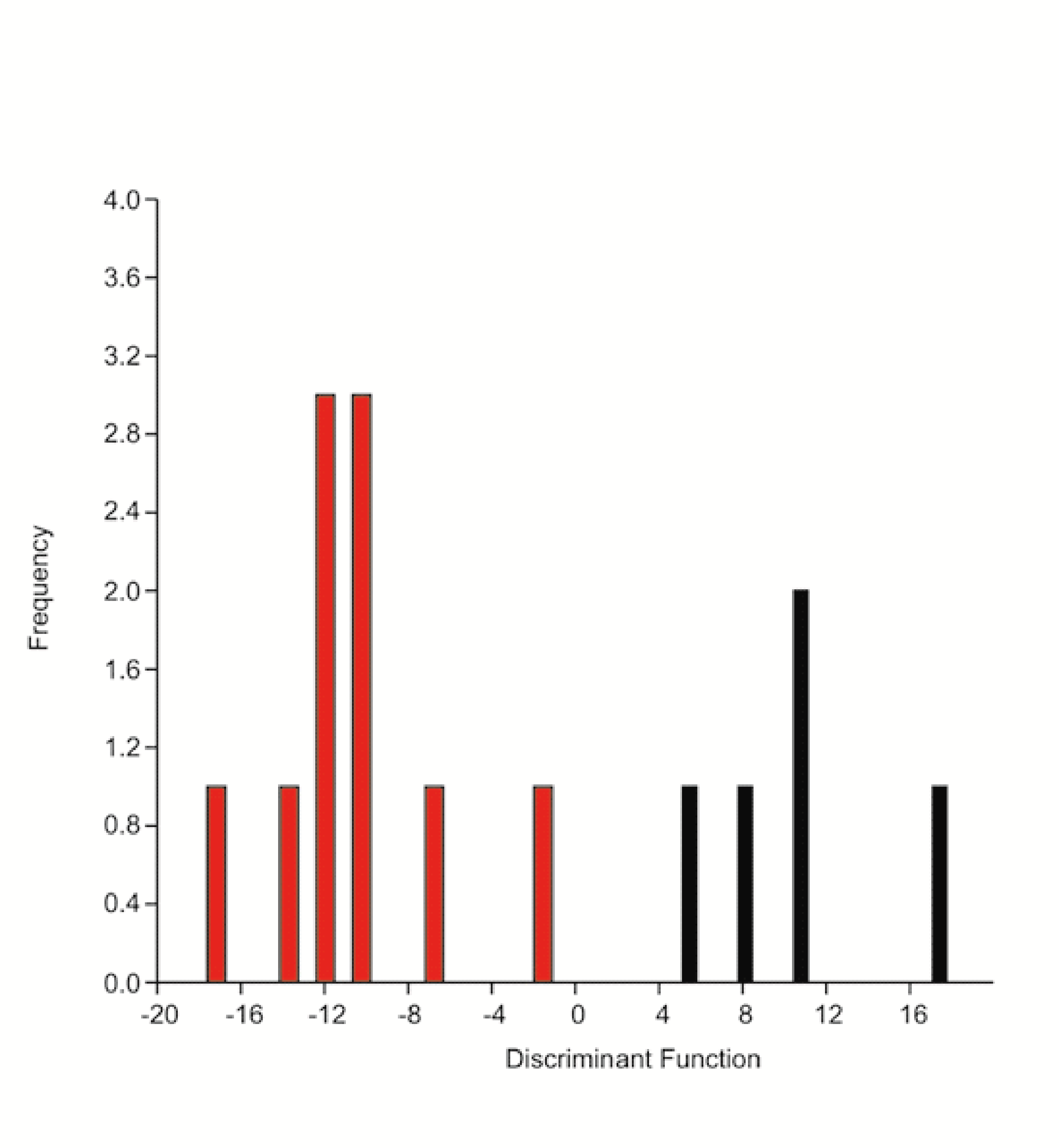

Supplement: S4 Fig — Black: group S01 (green line). Red: groups S02 and S03 (traditional line). (TIF) [file pone.0170281.s004.tif]

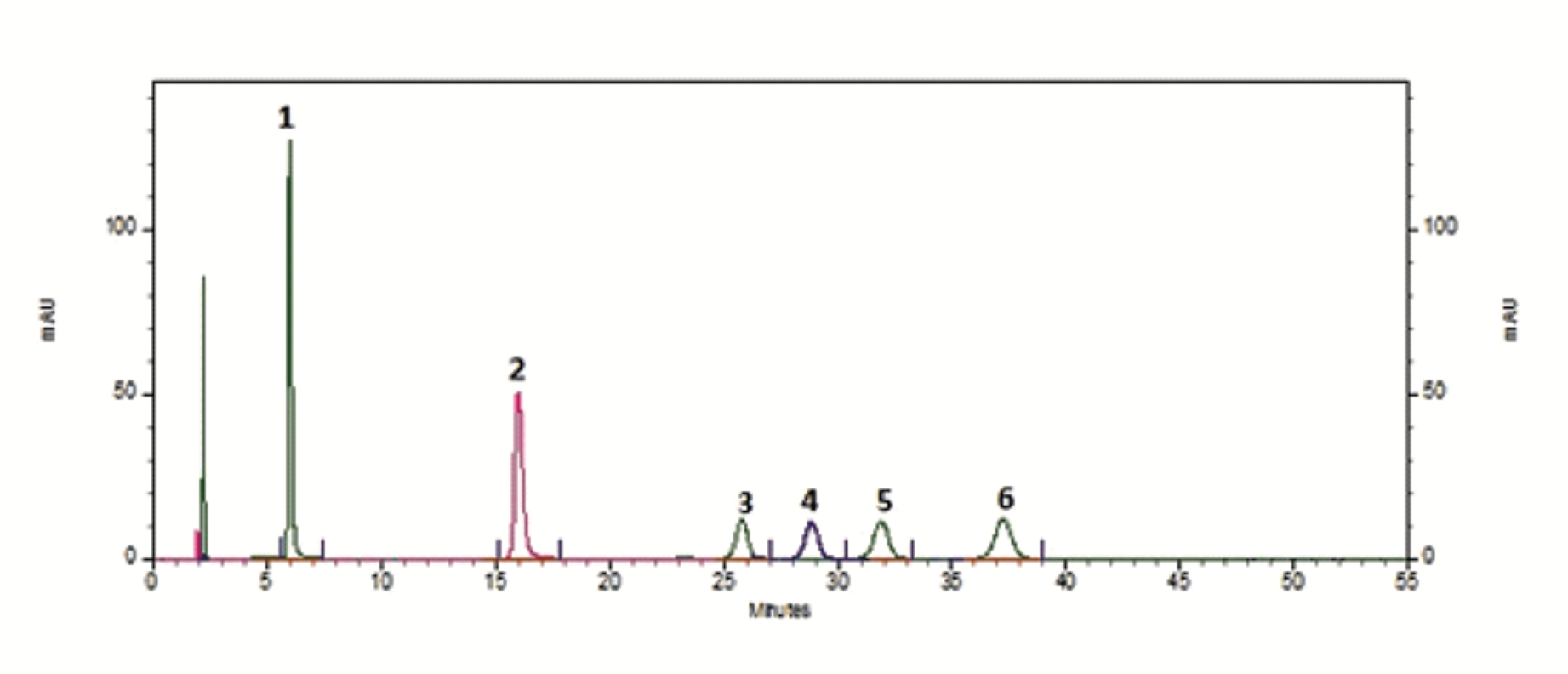

Supplement: S5 Fig — Peak number 1 (macaubine nitrate—0,02 mg/mL); peak number 2 (pilocarpine—0,05 mg/mL); peak number 3 (epiisopilosine—0,02 mg/mL); peak number 4 (epiisopiloturine—0,02 mg/mL); peak number 5 (isopilosine—0,02 mg/mL); peak number 6 (pilosine—0,02 mg/mL). (TIF) [file pone.0170281.s005.tif]
