# Supplementary material for: Seasonal change in main alkaloids of jaborandi (Pilocarpus microphyllus Stapf ex Wardleworth), an economically important species from the Brazilian flora
Source: PLoS One. 2017 Feb 2;12(2):e0170281. doi: 10.1371/journal.pone.0170281 (PMC5289444; doi:10.1371/journal.pone.0170281)
Supplement: S1 Table — (PI) Precipitation; (T) Temperature; (H) Humidity. (DOCX) [file pone.0170281.s006.docx]

**S1 Table**

|  |  | |  | |  |  | | | |  | |  | | | **Season** | | |  |  | | | | | | | | |  | |  | | |  |
| --- | --- | --- | --- | --- | --- | --- | --- | --- | --- | --- | --- | --- | --- | --- | --- | --- | --- | --- | --- | --- | --- | --- | --- | --- | --- | --- | --- | --- | --- | --- | --- | --- | --- |
|  |  | |  | |  | **Rainy** | | | |  | |  | | |  | | |  | **Dry** | | | | | | | | |  | |  | | |  |
|  | **Jan.** | **Feb.** | | **Mar.** | | | | **Apr.** | | | **May** | | | **Jun.** | | | **Jul.** | **Aug.** | | **Sept.** | | | **Oct.** | | | **Nov.** | | | | | **Dec.** | | |
| **PI (mm)** | 115.8 | 66.5 | | 155.7 | | | 160.5 | | 130.0 | | | | 101.0 | | | 38.3 | | - | | | - | | | - | | | - | | | | | - | |
| **T (ºC)** | 32.0 | 33.1 | | 34.2 | | | 34.2 | | 34.9 | | | | 34.2 | | | 33.8 | | 35.2 | | | 32.4 | 31.0 | | | 30.2 | | | | 33.8 | | | | |
| **H(%)** | 56 | 66 | | 63 | | | 64 | | 59 | | | | 58 | | | 50 | | 43 | | | 53 | | | 55 | | | 70 | | | | | 56 | |
