# Supplementary material for: Seasonal change in main alkaloids of jaborandi (Pilocarpus microphyllus Stapf ex Wardleworth), an economically important species from the Brazilian flora
Source: PLoS One. 2017 Feb 2;12(2):e0170281. doi: 10.1371/journal.pone.0170281 (PMC5289444; doi:10.1371/journal.pone.0170281)
Supplement: S2 Table — Data represents the mean values ± standard deviation. a No. LComm Bands: no. of Locally Common Bands; b h: diversity;c uh: unbiased diversity (DOCX) [file pone.0170281.s007.docx]

**S2 Table**

| **Population** | **S01** | **S02** | **S03** |
| --- | --- | --- | --- |
| **No. Bands** | 89 | 86 | 99 |
| **No. Bands Frequent ≥ 5%** | 89 | 86 | 99 |
| **No. Private Bands** | 5 | 1 | 11 |
| **No. LComm^a^ Bands ≤ 25%** | 0 | 0 | 0 |
| **No. LComm^a^ Bands ≤ 50%** | 0 | 0 | 0 |
| **Mean h^b^** | 0.203 ± 0.019 | 0.199 ± 0.020 | 0.274 ± 0.019 |
| **Mean uh^c^** | 0.254 ± 0.024 | 0.249 ± 0.024 | 0.342 ± 0.024 |
