# Supplementary material for: Seasonal change in main alkaloids of jaborandi (Pilocarpus microphyllus Stapf ex Wardleworth), an economically important species from the Brazilian flora
Source: PLoS One. 2017 Feb 2;12(2):e0170281. doi: 10.1371/journal.pone.0170281 (PMC5289444; doi:10.1371/journal.pone.0170281)
Supplement: S3 Table — (DOCX) [file pone.0170281.s008.docx]

**S3 Table**

| **Alkaloid** | **Structure** | **Structural Data** |
| --- | --- | --- |
| Epiisopiloturine | 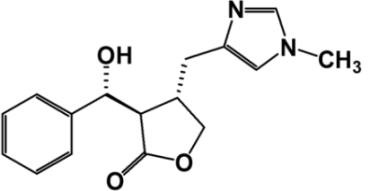 | The ^1^H NMR spectrum of the EPI shows the presence of the H from the CH_3_ group (C16) at 3 ppm. It is observed the difference between the H (C9) at 5.30 ppm and the H (C4) at around 2.3 ppm due to the deshielding of the H from the hydroxyl group. Moreover, the H from the imidazole and benzene aromatic ring at 7 ppm is deshielded by the resonance. The H (C6 and C7) is slightly more deshielded than the H (C5 and C8) due to the lactone group. The ^13^C NMR spectrum of the EPI presents the peaks related to the CH_3_ and CH_2_ groups (C16 and C4) at 32-31 ppm and CH (C9) at 71 ppm, where the latter is deshielded by the hydroxyl group compared to the C4, likewise was observed for the ^1^H NMR spectrum. The C1 and C3 from the imidazole ring are deshielded due to the resonance of the aromatic ring. Additionally, the resonance peaks at 130-133 ppm related to the CH (C11, C12, C13, C14 and C15) of the benzene ring are slightly less deshielded than the C1 and C3 from the imidazole aromatic ring. Finally, downfield is observed for the C7, from the lactone ring, that is deshielded due to the linkage to the oxygen atom, whereas the CH_2_ (C6) directly joined to the C-O (lactone group) and C8 (CH) are shielded by the ring. |
| Epiisopilosine | 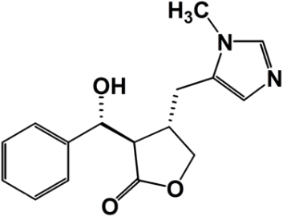 | ^1^H NMR (600.13 MHz, CD_3_OD+CDCl_3_, ppm): *δ* 7.32 (m, 4H, H11,15 + H12,14), 7.27 (s, 1H, H1), 7.25 (m, 1H, H13), 6.54 (d, *J* 0.5 Hz, 1H, H2), 5.34 (d, *J* 2.2 Hz, 1H, H9), 4.45 (dd, *J* 8.8, 7.6 Hz, 1H, H6a), 4.02 (dd, *J* 8.8, 4.4 Hz, 1H, H6b), 3.14 (s, 3H, NCH_3_), 2.78 (dd, *J* 2.7, 2.2 Hz, 1H, H8), 2.78 (m, 1H, H5), 2.54 (dd, *J* 15.2, 7.9 Hz, 1H, H4a), 2.21 (dd, *J* 15.2, 6.7 Hz, 1H, H4b). ^13^C NMR (150.90 MHz, CD_4_OD+CDCl_3_, ppm): *δ* 180.0 (C7), 143.2 (C10), 139.0 (C1), 129.6 (C3), 129.3 (C12,14), 128.1 (C13), 127.1 (C2), 126.0 (C11,15), 74.0 (C6), 72.1 (C9), 54.1 (C8), 34.3 (C5), 31.3 (NCH_3_), 28.8 (C4). |
| Isopilosine | 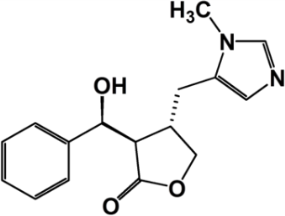 | ^1^H NMR (600.13 MHz, CD_3_OD, ppm): *δ* 7.49 (s, 1H, H1), 7.38 (dd, *J* 7.2, 1.6 Hz, 2H, H11,15), 7.34 (t, *J* 7.2 Hz, 2H, H12,14), 7.29 (tt, *J* 7.2, 1.6 Hz, 1H, H13), 6.71 (d, *J* 0.7 Hz, 1H, H2), 5.04 (d, *J* 5.5 Hz, 1H, H9), 4.02 (dd, *J* 9.2, 7.3 Hz, 1H, H6a), 3.90 (dd, *J* 9.2, 5.9 Hz, 1H, H6b), 3.46 (s, 3H, NCH_3_), 2.89 (dd, *J* 6.5, 5.5 Hz, 1H, H8), 2.70 (d, *J* 9 Hz, 1H, H4a), 2.70 (d, *J* 6.5 Hz, 1H, H4b), 2.66 (m, 1H, H5). ^13^C NMR (150.90 MHz, CD_4_OD, ppm): *δ* 179.0 (C7), 142.8 (C10), 139.5 (C1), 130.5 (C3), 129.5 (C12,14), 129.1 (C13), 127.6 (C11,15), 127.1 (C2), 73.9 (C9), 72.9 (C6), 54.0 (C8), 37.6 (C5), 31.7 (NCH_3_), 28.2 (C4). |
| Pilosine | 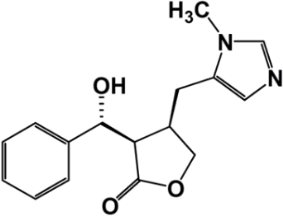 | ^1^H NMR (600.13 MHz, CD_3_OD, ppm): *δ* 7.52 (d, *J* 7.8 Hz, 2H, H11,15), 7.45 (s, 1H, H1), 7.35 (dd, *J* 7.8, 7.5 Hz, 2H, H12,14), 7.25 (t, *J* 7.5 Hz, 1H, H13), 6.69 (s, 1H, H2), 5.22 (d, *J* 5.9 Hz, 1H, H9), 4.21 (dd, *J* 9.0, 5.9 Hz, 1H, H6a), 4.13 (dd, *J* 9.0, 3.4 Hz, 1H, H6b), 3.49 (dd, *J* 7.6, 5.9 Hz, 1H, H8), 3.34 (s, 3H, NCH_3_), 2.96 (dd, *J* 15.4, 3.7 Hz, 1H, H4a), 2.86 (m, 1H, H5), 2.64 (d, *J* 15.4,11.7 Hz, 1H, H4b). ^13^C NMR (150.90 MHz, CD_4_OD, ppm): *δ* 179.0 (C7), 144.3(C10), 139.3 (C1), 131.5 (C3), 129.3 (C12,14), 128.3 (C13), 127.3 (C11,15), 126.7 (C2), 71.9 (C9), 71.7 (C6), 50.7 (C8), 38.8 (C5), 31.4(NCH_3_), 23.4 (C4). |
